# Association between depression and anxiety and inability to achieve remission in rheumatoid arthritis and psoriatic arthritis

**DOI:** 10.1093/rheumatology/keae621

**Published:** 2024-11-06

**Authors:** Selinde V J Snoeck Henkemans, Marijn Vis, Gonul Hazal Koc, Jolanda J Luime, Marc R Kok, Ilja Tchetverikov, Sjoerd M van der Kooij, Jessica Bijsterbosch, Annette H M van der Helm-van Mil, Pascal H P de Jong

**Affiliations:** Department of Rheumatology, Erasmus MC, Rotterdam, The Netherlands; Department of Rheumatology, Erasmus MC, Rotterdam, The Netherlands; Department of Rheumatology, Erasmus MC, Rotterdam, The Netherlands; Department of Rheumatology, Erasmus MC, Rotterdam, The Netherlands; Department of Rheumatology, Maasstad Hospital, Rotterdam, The Netherlands; Department of Rheumatology, Albert Schweitzer Hospital, Dordrecht, The Netherlands; Department of Rheumatology, Haga Hospital, The Hague, The Netherlands; Department of Rheumatology, Amphia Hospital, Breda, The Netherlands; Department of Rheumatology, Erasmus MC, Rotterdam, The Netherlands; Department of Rheumatology, Leiden University Medical Centre, Leiden, The Netherlands; Department of Rheumatology, Erasmus MC, Rotterdam, The Netherlands

**Keywords:** rheumatoid arthritis, psoriatic arthritis, remission, depression, anxiety

## Abstract

**Objectives:**

To investigate the association between depression and anxiety and the inability to achieve remission in RA and PsA patients. In addition, the association between depressive and anxiety symptoms and disease activity components was explored.

**Methods:**

A total of 400 RA and 367 PsA patients from the tREACH and DEPAR were included, respectively. Patients had a possible depression or anxiety disorder if they scored >7 on the Hospital Anxiety and Depression Scale (HADS). Remission was defined as DAS44 <1.6 in RA and DAPSA ≤ 4 in PsA. Mixed models were used to assess the association between depression/anxiety, at any timepoint during 2 years, and remission during 2 years, and to explore which disease activity components are most influenced by depression/anxiety.

**Results:**

At baseline, 20% of RA patients had a possible depression and 30% a possible anxiety disorder. In PsA this was 18% and 23%. After adjustment for concurrent anxiety symptoms, depression was associated with a lower odds of achieving remission during 2 years of follow-up [OR 0.45 (95%CI 0.25–0.80) for RA and OR 0.24 (95%CI 0.08–0.71) for PsA]. Anxiety was not associated with remission after adjustment for concurrent depression symptoms. The presence of depression/anxiety was associated with higher tender joint count, worse general health, more pain and slightly elevated inflammation markers, but not with more swollen joints in both RA and PsA.

**Conclusion:**

The presence of depressive symptoms in RA and PsA patients at baseline or during follow-up was associated with a lower likelihood of achieving remission. Healthcare professionals should, therefore, be aware of symptoms of depression.

Rheumatology key messages

- Both depression and anxiety are associated with a lower likelihood of remission in RA and PsA, although anxiety is not significant in adjusted analyses.
- Patients with a possible depression or anxiety disorder have more tender joints, worse general health and slightly elevated inflammatory markers.
- Recognizing a possible depression is important for optimal disease management in RA and PsA.

## Introduction

In the treatment of RA and PsA, remission is the target to aim for [1, 2]. However, not all patients achieve remission [3]. The inability to achieve remission may have several causes. Reasons include delays in diagnosis and the presence of comorbidities, including depression and anxiety [4–6].

Depression and anxiety are common comorbidities in RA and PsA. A recent nationwide Danish study reported that ∼19% of RA patients and ∼27% of PsA patients suffer from depressive symptoms, and 22% of RA and 32% of PsA patients experience anxiety symptoms [7]. In contrast, in the general Danish population the prevalence of depression and anxiety was 9% and 11% in 2021, respectively [8]. However, prevalence estimates vary widely due to the use of different (self-reported) measures and cut-off points.

Several studies have suggested a bidirectional relationship between depression/anxiety and inflammatory arthritis [9–11]. Symptoms of depression and anxiety are associated with increased disease activity and vice versa [12–17]. This may be due to behavioural changes in patients with depression/anxiety, leading to reduced physical activity, fewer endorphins and increased sensitivity to pain. This, combined with repetitive negative thinking, may cause patients to score worse on patient-reported outcomes (PROs) [12]. Furthermore, depression appears to reduce the efficacy of treatment with DMARDs in RA, which may be partly due to decreased treatment adherence [12]. This association was not found for anxiety [18].

To date, depression and anxiety have mostly been explored as baseline predictors for poor outcomes. Moreover, most studies focused on RA patients [13, 14, 16, 17]. In addition, in some studies general measures of depression/anxiety were used (e.g. a single question from the EuroQol-5D or the SF-36) that do not distinguish between depression and anxiety [15–17, 19]. Studies exploring the effect of depression and anxiety separately, using a validated questionnaire, on remission over time in both RA and PsA are lacking [20]. Therefore, we aimed to investigate the association between symptoms of depression or anxiety, at any timepoint during 2 years follow-up, and the inability to achieve remission during follow-up in RA and PsA patients. In addition, the effect of depressive or anxiety symptoms on the disease activity components was explored.

## Methods

### Patients and study design

Data from the treatment in the Rotterdam Early Arthritis CoHort (tREACH) trial and the Dutch southwest Early Psoriatic Arthritis cohort (DEPAR) were used.

The tREACH was a multicentre, stratified, single-blinded randomized controlled trial. Eligible patients had arthritis in ≥1 joints and a symptom duration of <1 year. For this study, RA patients who fulfilled the 1987 and/or 2010 classification criteria for RA were selected (*n* = 425) [21, 22].

The DEPAR is a multicentre, prospective cohort study that started in 2013 and is ongoing [23]. Eligible patients have a new diagnosis of PsA, according to the treating rheumatologist, and have not yet had DMARDs for musculoskeletal inflammation. From DEPAR, data up to March 2019 were used from all consecutive PsA patients who were included between November 2013 and March 2017 (*n* = 442), to allow 2 years of follow-up. Patient selection was based on the availability of data from the Hospital Anxiety and Depression Scale (HADS).

The tREACH and DEPAR were both approved by the medical ethics committee of the Erasmus MC, the Netherlands (MEC-2006–252 for tREACH and MEC-2012–549 for DEPAR). All participants gave written informed consent before inclusion. Written informed consent was obtained from all participants according to the Declaration of Helsinki. Further details on the tREACH and DEPAR can be found elsewhere [5, 23, 24].

### Data collection

In the tREACH, patients were seen every 3 months during 2 years, and in DEPAR patients were seen every 3 months in the first year and every 6 months in the second year. At each visit patients were assessed by a trained research nurse, and clinical outcomes and blood samples were collected. Clinical outcomes included a 44-swollen joint count (SJC44) and 53-tender joint count (TJC53) for RA patients and a SJC66, TJC68, psoriasis assessment (Body Surface Area; BSA), enthesitis assessment (Leeds Enthesitis Index; LEI) and dactylitis count for PsA patients [25, 26]. Blood samples included a CRP and ESR for both RA and PsA.

In addition, patients filled out questionnaires that captured the following PROs: pain, general health, depression and anxiety. Pain and general health were measured on a visual analogue scale (VAS), ranging from 0 to 100 millimeters (mm). Depression and anxiety were measured with the HADS [27]. The HADS was administered every 6 months in RA and every year in PsA and consists of a depression subscale (HADS-D) and an anxiety subscale (HADS-A). Scores range from 0 to 21 per subscale, and a score > 7 on the depression subscale or anxiety subscale suggests a possible depression or anxiety disorder, respectively [28]. In this study, depression and anxiety were considered separately and clinically relevant symptoms of depression or anxiety were defined as a score >7 on the HADS-D or HADS-A, respectively. Patients who score >7 on the HADS-D will be referred to as ‘depressed’ and patients who score >7 on the HADS-A will be referred to as ‘anxious’ in this article.

### Disease activity

In RA, disease activity was assessed with the DAS44 which includes a SJC44, TJC53, ESR (in mm/h) and general health (VAS, 0–100mm). The DAS44 threshold for remission is <1.6 [29].

In PsA, the Disease Activity index for Psoriatic Arthritis (DAPSA) was used to measure disease activity. The DAPSA was calculated by taking the sum of the SJC66, TJC68, CRP (in mg/dl), general health (VAS, 0–100mm divided by 10), and pain (VAS, 0–100mm divided by 10). The DAPSA threshold for remission is ≤4 [30]. These composite disease activity measures were chosen because current treat-to-target guidelines recommend the use of the DAS for RA and the DAPSA for PsA to assess disease activity [31, 32].

### Statistical analysis

To examine differences in patient characteristics between patients with and without depression/anxiety at baseline, an independent *t* test, Mann–Whitney *U* test or X^2^ test were used, when appropriate.

For the primary outcome, patients with and without depression or anxiety, at any timepoint during 2 years follow-up, were compared on their ability to achieve remission, using mixed logit models with an unstructured covariance matrix. The unadjusted model included time (months) and a random intercept (individual patients). Depression and anxiety are common comorbidities and we aimed to assess depression and anxiety separately [33]. Therefore, we first added the presence of anxiety (HADS-A score >7) in the adjusted analyses of depression, and the presence of depression (HADS-D score >7) in the adjusted analyses of anxiety. Thereafter, we performed a fully adjusted model in which additional corrections were made for sex, symptom duration, smoking (yes/no) and baseline DAS44 and randomization strata (in RA) or baseline DAPSA and the presence of enthesitis (in PsA).

For the secondary outcome, the mean values of the DAS44 (RA) and DAPSA (PsA) during 2 years of follow-up were compared between patients with and without depression or anxiety, at any timepoint, using linear mixed models (LMM). The DAS44/DAPSA components were the dependent variables and anxiety (yes/no; in the depression analysis) or depression (yes/no; in the anxiety analysis), time (months), sex, symptom duration, smoking (yes/no), a random intercept and randomization strata (in RA) or the presence of enthesitis (in PsA) were the independent variables in the LMMs.

The completeness rate of the HADS is reported in Supplementary Table S1, available at *Rheumatology* online. After 2 years of follow-up, 60% and 67% of the RA and PsA patients still filled out their HADS questionnaire, respectively. Missing data were handled with the use of mixed models.

Finally, we performed three sensitivity analyses. Firstly, the continuous HADS score instead of the dichotomized score was used to assess the effect of a 1-unit change in HADS score on the ability to achieve remission. Secondly, the DAS44-3 item, that is, the DAS44 without general health, was used for the definition of remission in RA to evaluate circularity in the results due to possibly higher scores on general health in those with depression/anxiety. Finally, the analyses in PsA were repeated in patients with an oligo- or poly-arthritis to rule out enthesitis as the main driving factor of the effect.

A *P*-value ≤ 0.05 was considered statistically significant. Analyses were performed in Stata v18.0.

## Results

### Patients

Of the 425 included RA patients, 400 (94%) had data on depression/anxiety at baseline. From these 400 RA patients, 78 (20%) were depressed and 120 (30%) were anxious. At baseline, RA patients with depression or anxiety had more tender joints and a worse general health than those without depression/anxiety and thus had a higher DAS44. RA patients with anxiety also had a longer symptom duration (Table 1). A total of 51 (13%) RA patients scored >7 on both the HADS-D and HADS-A at baseline.

**Table 1.** Baseline characteristics of RA and PsA patients with and without a possible depression or anxiety disorder, according to the HADS

	Rheumatoid arthritis			
	Depressed	Not depressed	Anxious	Not anxious
	*n* = 78	*n* = 322	*n* = 120	*n* = 280
Demographic characteristics				
Age (years), mean (SD)	52 (15)	55 (14)	52 (15)	55 (14)
Sex (female), *n* (%)	53 (68)	213 (66)	83 (69)	183 (65)
Symptom duration (months), median (IQR)	5 (3–8)	5 (3–7)	5 (4–8)	4 (3–7)^a^
Currently smoking, *n* (%)	28 (36)	92 (29)	42 (35)	78 (28)
Body mass index (kg/m^2^), mean (SD)	25.5 (4.6)	26.4 (4.8)	26.3 (4.5)	26.2 (4.9)
Disease activity				
DAS44, mean (SD)	3.6 (1.0)	3.2 (0.9)^a^	3.6 (1.0)	3.2 (0.9)^a^
Swollen joint count (44), median (IQR)	8 (4–12)	7 (4–12)	7 (4–12)	8 (4–12)
Tender joint count (53), median (IQR)	13 (6–20)	9 (4–14)^a^	12 (7–17)	8 (4–14)^a^
ESR (mm/h), median (IQR)	25 (13–42)	20 (12–38)	23 (11–40)	20 (12–38)
General health (VAS), median (IQR)	63 (49–74)	50 (29–65)^a^	53 (40–70)	50 (28–65)^a^

	Psoriatic arthritis			
	Depressed	Not depressed	Anxious	Not anxious
	*n* = 67	*n* = 300	*n* = 85	*n* = 282
Demographic characteristics				
Age (years), mean (SD)	49 (14)	51 (13)	49 (14)	51 (13)
Sex (female), *n* (%)	43 (64)	138 (46)^a^	57 (67)	124 (44)^a^
Symptom duration (months), median (IQR)	9 (4–38)	12 (4–32)	12 (4–38)	10 (3–31)
Currently smoking, *n* (%)	31 (47)	53 (18)^a^	30 (36)	54 (19)^a^
Body mass index (kg/m^2^), mean (SD)	28.9 (5.5)	28.0 (4.9)	28.5 (5.2)	28.1 (5.0)
Disease activity				
DAPSA, median (IQR)	22 (17–31)	14 (9–21)^a^	20 (14–28)	14 (9–21)^a^
Swollen joint count (66), median (IQR)	2 (1–4)	2 (0–4)	1 (0–3)	2 (1–4)
Tender joint count (68), median (IQR)	5 (2–11)	2 (1–6)^a^	5 (2–9)	2 (1–6)^a^
CRP (mg/L), median (IQR)	5 (2–16)	4 (1–10)	5 (2–14)	4 (0–10)^a^
General health (VAS), median (IQR)	71 (52–81)	39 (20–57)^a^	68 (50–80)	39 (19–57)^a^
Pain (VAS), median (IQR)	71 (57–81)	44 (21–61)^a^	66 (48–76)	41 (21–62)^a^
Psoriasis, *n* (%)	60 (90)	259 (86)	76 (89)	243 (86)
BSA (%) in case of psoriasis, median (IQR)	3 (1–6)	3 (1–5)	3 (1–6)	3 (1–5)
Enthesitis, *n* (%)	36 (54)	108 (36)^a^	45 (53)	99 (35)^a^
LEI in case of enthesitis, median (IQR)	2 (1–4)	2 (1–3)	2 (1–4)	2 (1–3)
Dactylitis, *n* (%)	9 (13)	52 (17)	12 (14)	49 (17)

a Indicates a statistical significant difference between patients with and without a possible depression, or with and without a possible anxiety disorder at baseline.

BSA: body surface area; DAPSA: Disease Activity index for Psoriatic Arthritis; DAS44: 44-joint Disease Activity Score; HADS: Hospital Anxiety and Depression Scale; IQR: interquartile range; LEI: Leeds Enthesitis Index; VAS: Visual Analogue Scale.

Of the 442 included PsA patients, 367 (83%) had data on depression/anxiety at baseline. From these 367 PsA patients, 67 (18%) were depressed and 85 (23%) were anxious according to the HADS >7. PsA patients with depression or anxiety were more often female, smokers and more often presented with enthesitis. They also had more tender joints, a worse general health and more pain than those without depression/anxiety and thus had a higher DAPSA. PsA patients with anxiety also had slightly higher CRP levels at baseline (Table 1). In total, 49 (13%) PsA patients scored >7 on both the HADS-D and HADS-A at baseline.

### Baseline depression/anxiety and achieving remission after 1 and 2 years

**Figure 1.**
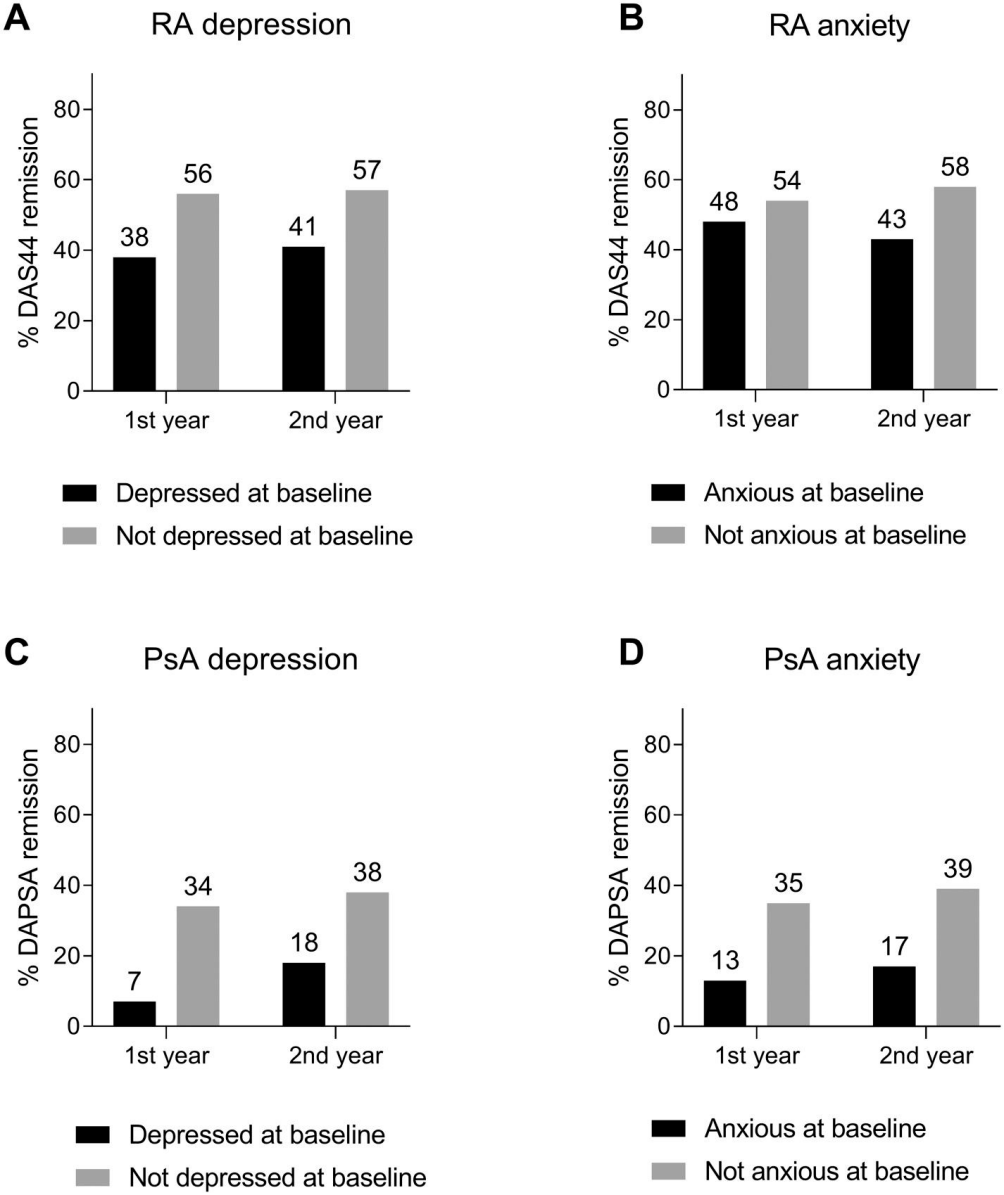
Proportion of RA and PsA patients in remission at 1 and 2 years, stratified for the presence or absence of depression or anxiety at baseline. (**A**) and (**B**) show RA patients with and without a possible depression or anxiety disorder at baseline, defined as a HADS-D or HADS-A >7, respectively. (**C**) and (**D**) show PsA patients with and without a possible depression or anxiety disorder at baseline, defined as a HADS-D or HADS-A >7, respectively. DAPSA: Disease Activity index for Psoriatic Arthritis; DAS44: 44-joint Disease Activity Score; HADS-A: Hospital Anxiety and Depression Scale – Anxiety subscale; HADS-D: Hospital Anxiety and Depression Scale – Depression subscale

Remission was less often achieved after 1 and 2 years of follow-up in RA as well as PsA patients with depression or anxiety at baseline (Fig. 1). In RA the largest difference in remission rate was seen after 1 year of follow-up in patients with and without depression at baseline (38% *vs* 56%). Similar results were found in PsA (7% *vs* 35%).

### Depression/anxiety variance during 2 years of follow-up

**Figure 2.**
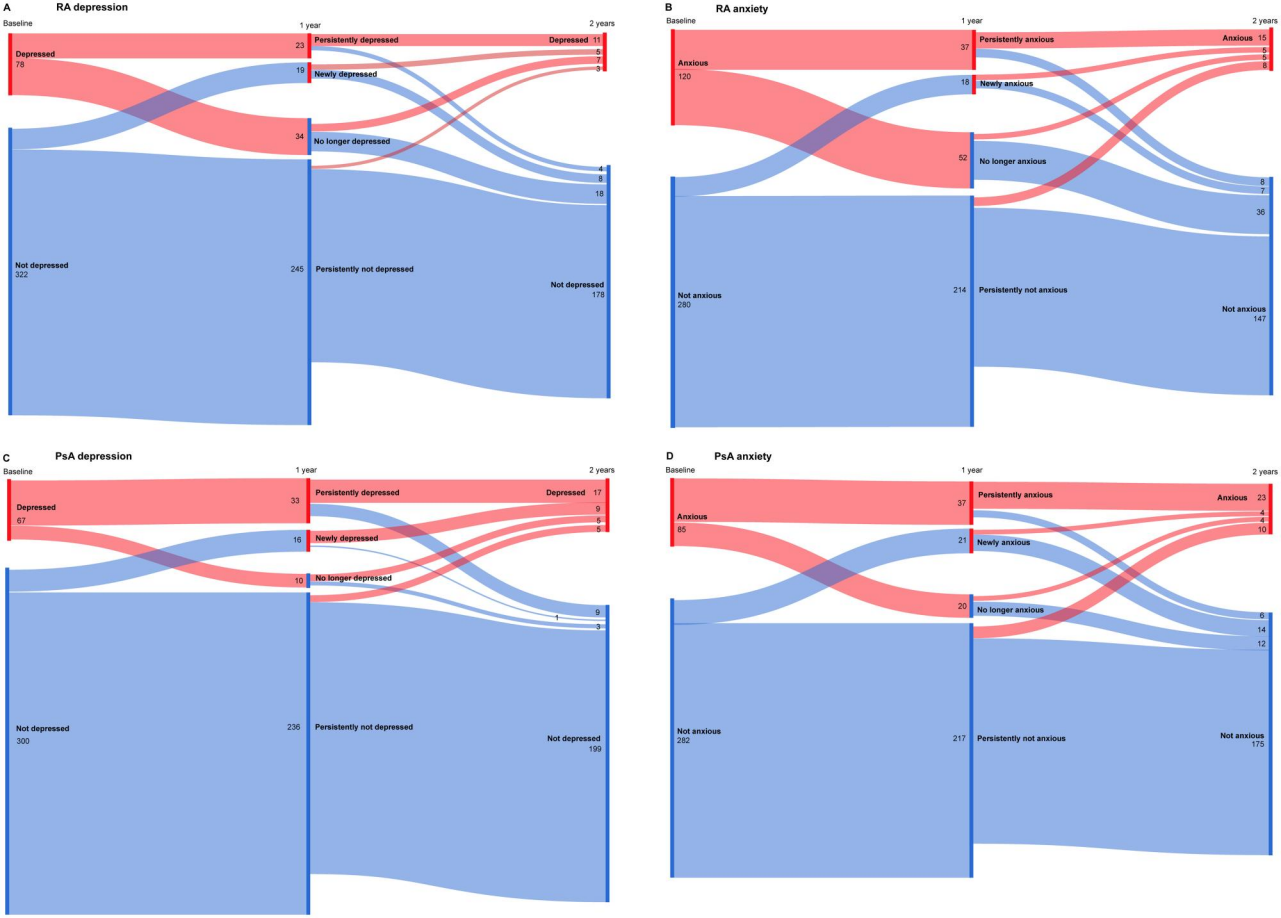
Patient flows during 2 years of follow-up for RA and PsA patients with and without depression or anxiety at baseline. (**A**) and (**B**) show RA patients with and without a possible depression or anxiety disorder at baseline, defined as a HADS-D or HADS-A >7 and ≤7, respectively. (**C**) and (**D**) show PsA patients with and without a possible depression or anxiety disorder at baseline, defined as a HADS-D or HADS-A >7 and ≤7, respectively. HADS-A: Hospital Anxiety and Depression Scale – Anxiety subscale; HADS-D: Hospital Anxiety and Depression Scale – Depression subscale

The proportion of RA patients with depression/anxiety varied over time (Fig. 2). For example, 40% of RA patients with depression at baseline were also depressed after 1 year of follow-up and 73% of those patients were still depressed after 2 years. The proportion of RA patients without depression remained relatively stable over time. Similar trends were found for RA patients with anxiety (Fig. 2).

RA patients who were not depressed at baseline, 1 and 2 years had a lower median HADS at baseline (median 1, IQR 0–2), than those who were not depressed at baseline but became depressed after 1 year (median 5, IQR 2–6). Patients who remained not anxious at baseline, 1 and 2 years also had a lower HADS at baseline (median 3, IQR 2–5) compared with those who became anxious after 1 year (median 5, IQR 4–6).

RA patients who were depressed/anxious at baseline, but not after 1 year of follow-up had a greater improvement in DAS44 compared with patients who were persistently depressed/anxious. In contrast, RA patients who were not depressed/anxious at baseline, but became depressed/anxious after 1 year had a larger DAS44 improvement than patients who were persistently depressed/anxious (Supplementary Table S2, available at *Rheumatology* online).

Of the PsA patients with depression at baseline, 77% still had depressive symptoms after 1 year and 65% of those patients also had depressive symptoms after 2 years. Similarly to RA, the proportion of PsA patients without depression remained relatively stable over time. Similar trends were found for PsA patients with anxiety (Fig. 2).

Patients who were persistently not depressed had a lower HADS at baseline (median 2, IQR 1–4), than those who were not depressed at baseline but became depressed after 1 year (median 4.5, IQR 2–6.5). The same was true for those who were persistently not anxious [median HADS at baseline 3 (IQR 1–5)], *vs* those who became anxious after 1 year (median 6, IQR 4–7).

PsA patients who had a resolution of their depression/anxiety after 1 year of follow-up had a greater DAPSA improvement than patients who were continuously depressed/anxious. For anxiety, this difference was significant. PsA patients who were not depressed at baseline, but became depressed after 1 year of follow-up had a smaller DAPSA improvement compared with patients who were not depressed during the entire follow-up. In contrast, patients who became anxious after 1 year had a larger DAPSA improvement than those who were not anxious during follow-up (Supplementary Table S2, available at *Rheumatology* online).

### Depression/anxiety and achieving remission during 2 years of follow-up

**Figure 3.**
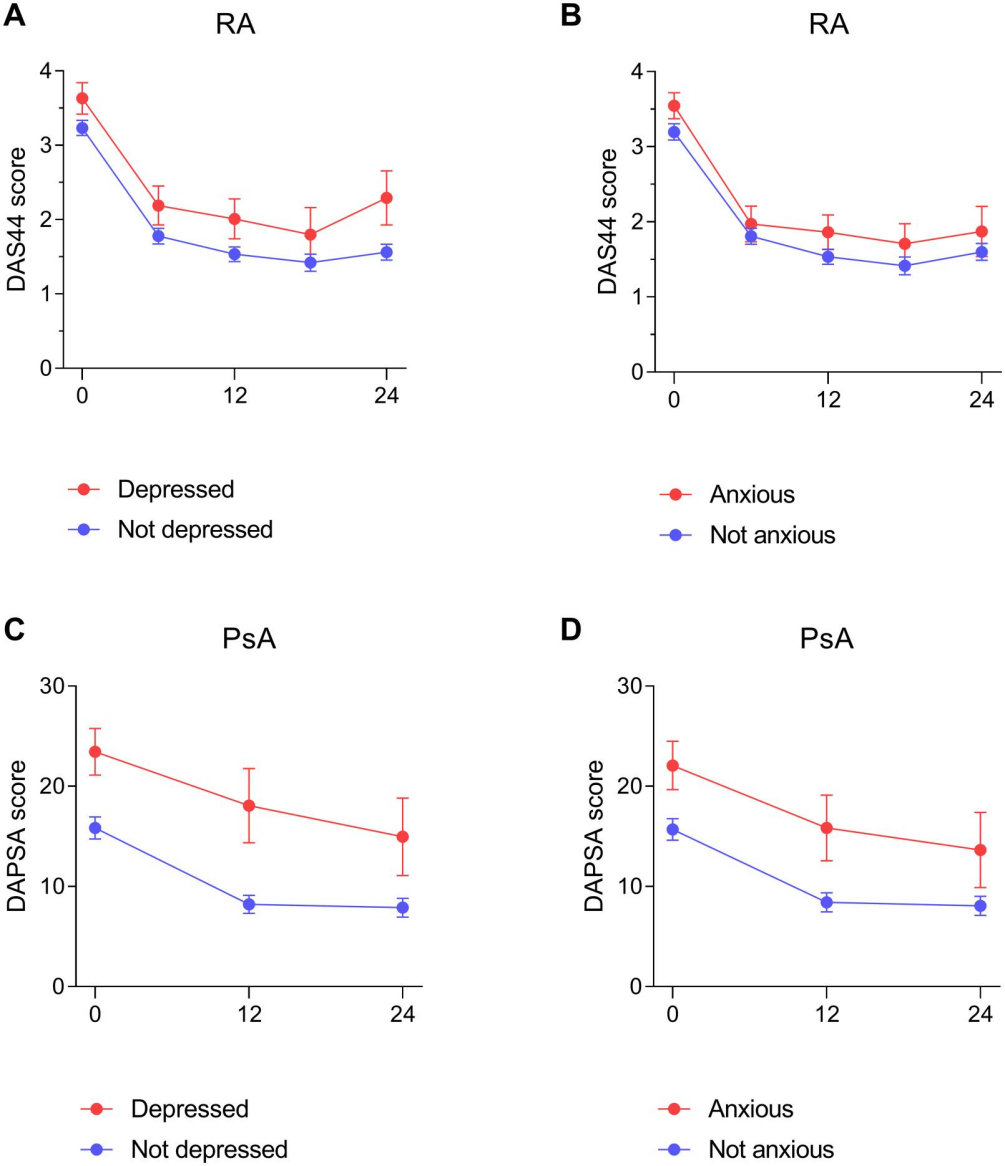
Mean (with 95% CI) DAS44 and DAPSA score during 2 years of follow-up in RA and PsA patients with and without depression or anxiety, at any timepoint during 2 years of follow-up. (**A**) and (**B**) show RA patients with and without a possible depression or anxiety disorder at any timepoint during 2 years of follow-up, defined as a HADS-D or HADS-A >7 and ≤7, respectively. (**C**) and (**D**) show PsA patients with and without a possible depression or anxiety disorder during 2 years of follow-up, defined as a HADS-D or HADS-A >7 and ≤7, respectively. DAPSA: Disease Activity index for Psoriatic Arthritis; DAS44: 44-joint Disease Activity Score; HADS-A: Hospital Anxiety and Depression Scale – Anxiety subscale; HADS-D: Hospital Anxiety and Depression Scale – Depression subscale

RA patients with depression/anxiety, at any timepoint, had higher mean DAS44 scores during 2 years of follow-up compared with those without depression/anxiety (Fig. 3). Similarly, PsA patients with depression/anxiety had higher mean DAPSA scores over 2 years than those without depression/anxiety (Fig. 3). RA patients who had both a possible depression and anxiety disorder, at any timepoint, had similar mean DAS44 scores compared with patients with only a depression, but they had higher mean DAS44 scores compared with patients with solely an anxiety disorder (Supplementary Fig. S1, available at *Rheumatology* online). Similarly, PsA patients who had both a possible depression and anxiety disorder had similar mean DAPSA scores as patients with a depression only, but they had higher mean DAPSA scores compared with patients who were only anxious (Supplementary Fig. S1, available at *Rheumatology* online).

**Figure 4.**
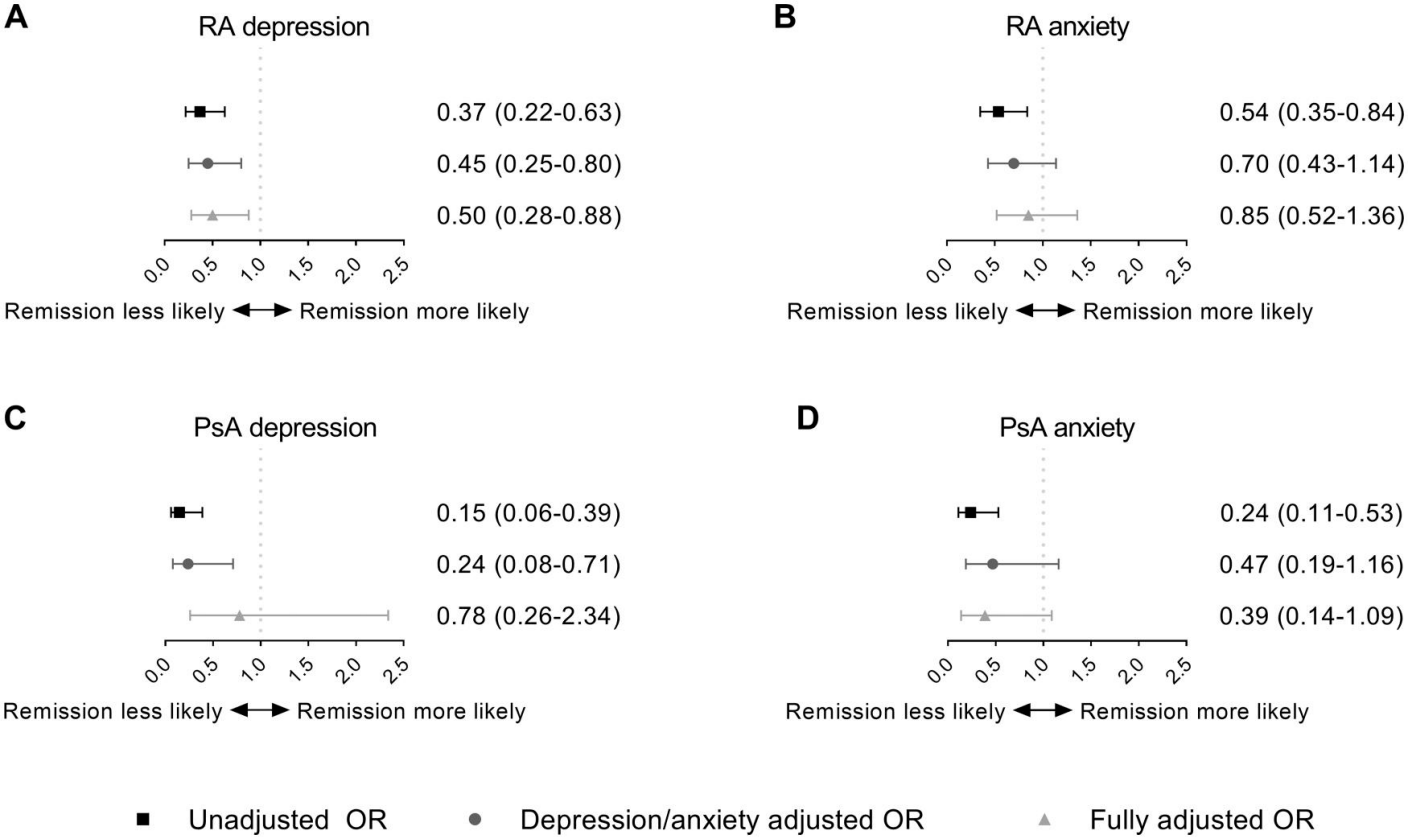
Odds of achieving remission over 2 years of follow-up in RA and PsA patients with depression or anxiety, at any timepoint during 2 years of follow-up. (**A**) and (**B**) show the unadjusted and adjusted ORs with 95% CI for the likelihood of achieving remission, defined as DAS44 <1.6, during 2 years of follow-up for RA patients with a possible depression or anxiety disorder at any timepoint, defined as a HADS-D or HADS-A >7, respectively. (**C**) and (**D**) show the unadjusted and adjusted ORs with 95% CI for the likelihood of achieving remission, defined as DAPSA ≤4, during 2 years for PsA patients with a possible depression or anxiety disorder at any timepoint, defined as a HADS-D or HADS-A >7, respectively. In both RA and PsA the adjusted OR was corrected for sex, symptom duration and smoking. In RA we also corrected for baseline DAS and randomization strata and in PsA for the baseline DAPSA and the presence of enthesitis. DAPSA: Disease Activity index for Psoriatic Arthritis; DAS44: 44-joint Disease Activity Score; HADS-A: Hospital Anxiety and Depression Scale – Anxiety subscale; HADS-D: Hospital Anxiety and Depression Scale – Depression subscale; OR: odds ratio

Depression/anxiety was associated with lower odds of achieving remission in both RA and PsA (Fig. 4). However, after adjustment for depression in the analyses of anxiety and vice versa, only depression remained associated with a lower likelihood of achieving remission. The depression/anxiety adjusted odds ratio (OR) for DAS44 remission was 0.45 (95%CI 0.25–0.80) in RA patients with depression and 0.70 (95%CI 0.43–1.14) in those with anxiety. After full adjustment, the ORs increased to 0.50 (95%CI 0.28–0.88) and 0.85 (95%CI 0.52–1.36) for depression and anxiety, respectively (Fig. 4). For PsA the depression/anxiety adjusted ORs for achieving DAPSA remission were 0.24 (95%CI 0.08–0.71) and 0.47 (95%CI 0.19–1.16) in patients with depression or anxiety, respectively. Similarly, after full adjustment, the ORs increased [depression OR 0.78 (95%CI 0.26–2.34) and anxiety OR 0.39 (95%CI 0.14–1.09)] (Fig. 4).

### Depression/anxiety and disease activity components during 2 years

**Figure 5.**
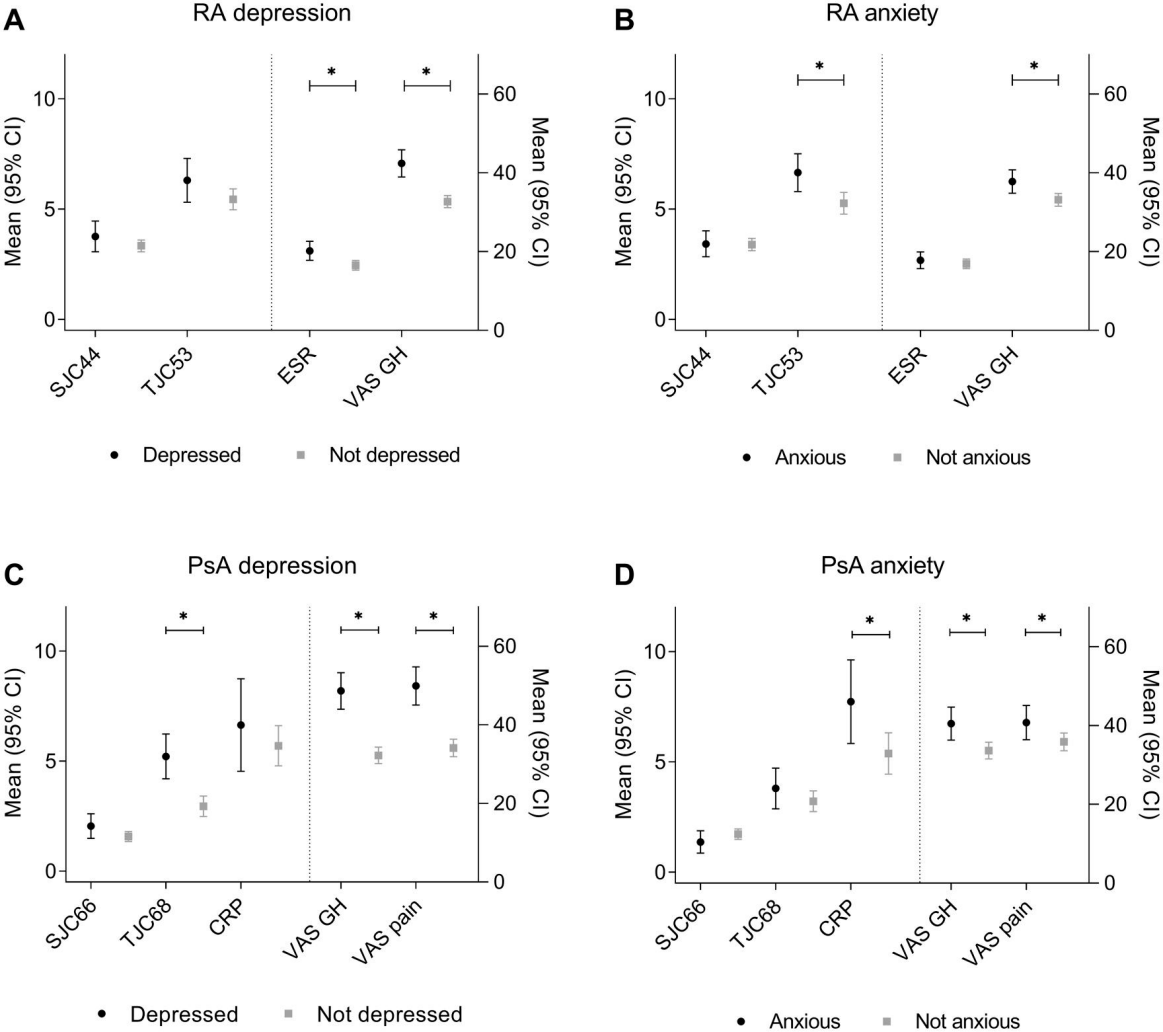
Predicted mean (with 95% CI) of the DAS44 and DAPSA components during 2 years of follow-up in RA and PsA patients with and without a depression or anxiety at any timepoint. (**A**) and (**B**) show RA patients with and without a possible depression or anxiety disorder, defined as a HADS-D or HADS-A >7 at any timepoint during 2 years of follow-up, respectively. (**C**) and (**D**) show PsA patients with and without a possible depression or anxiety disorder, defined as a HADS-D or HADS-A >7 at any timepoint during 2 years of follow-up, respectively. *Indicates a significant difference in the mean score of a DAS44/DAPSA component between patients with and without a possible depression or anxiety disorder, defined as a HADS-D or HADS-A >7 at any timepoint during 2 years of follow-up, respectively. DAPSA: Disease Activity index for Psoriatic Arthritis; DAS44: 44-joint Disease Activity Score; GH: general health; HADS-A: Hospital Anxiety and Depression Scale – Anxiety subscale; HADS-D: Hospital Anxiety and Depression Scale – Depression subscale; SJC44: 44-joint swollen joint count; SJC66: 66-joint swollen joint count; TJC53: 53-joint tender joint count; TJC68: 68-joint tender joint count; VAS: Visual Analogue Scale

The presence of depression/anxiety was associated with a worse general health in RA as well as PsA. In RA, depression was also associated with elevated ESR levels, and anxiety was associated with higher tender joint counts. In PsA, depression/anxiety was also associated with more pain. In addition, depression was associated with increased tender joint counts, and anxiety was associated with elevated CRP levels. However, we did not find an association between depression/anxiety and the number of swollen joints (Fig. 5).

### Sensitivity analyses

Drop-out rates were 26% for RA patients and 20% for PsA patients during 2 years of follow-up. Therefore, we compared baseline characteristics of patients who completed the follow-up with those who dropped out. RA patients who dropped out smoked more often, but did not otherwise differ from those who completed the follow-up (Supplementary Table S3, available at *Rheumatology* online). PsA patients who dropped out were younger, had poorer general health and more pain, but nevertheless had similar DAPSA scores at baseline (Supplementary Table S3, available at *Rheumatology* online).

If the continuous HADS instead of the dichotomized HADS was used in the mixed models, the odds of achieving remission during 2 years of follow-up in RA patients decreased with 20% (OR 0.80, 95%CI 0.76–0.86) per 1 point increase in the HADS depression score (Supplementary Fig. S2, available at *Rheumatology* online). The odds of remission decreased with 14% (OR 0.86, 95%CI 0.82–0.91) per point increase in the HADS anxiety score. For PsA, the odds of remission were 31% lower (OR 0.69, 95%CI 0.61–0.78) and 16% lower (OR 0.84, 95%CI 0.77–0.91) per point increase in HADS depression and anxiety score, respectively (Supplementary Fig. S2, available at *Rheumatology* online). After adjustment for confounders, including HADS-A/D score, the results were no longer significant for HADS-A, but remained significant for HADS-D.

If the DAS44-3 item instead of the original DAS44 was used in RA, depression and anxiety were still associated with a lower odds of achieving remission, but only in the unadjusted analyses; that is, unadjusted OR 0.56 (95%CI 0.35–0.89) and fully adjusted OR 0.74 (95%CI 0.45–1.24) for depression, and unadjusted OR 0.66 (95%CI 0.44–0.99) and adjusted OR 0.94 (95%CI 0.60–1.46) for anxiety (Supplementary Fig. S3, available at *Rheumatology* online). Finally, in PsA patients with an oligo-arthritis or poly-arthritis both the unadjusted and the anxiety-adjusted analysis showed that patients with depression had a lower odds of achieving remission during 2 years of follow-up [unadjusted OR 0.11 (95%CI 0.03–0.36) and anxiety-adjusted OR 0.11 (95%CI 0.03–0.43)]. However, the fully adjusted model for depression did not show a significant effect. In PsA patients with anxiety a significant effect was only observed in the unadjusted analysis [OR 0.35 (95%CI 0.14–0.86), while the depression-adjusted OR was 1.00 (95%CI 0.35–2.87)] (Supplementary Fig. S4, available at *Rheumatology* online).

## Discussion

This study investigated the association between symptoms of depression and anxiety at any timepoint during the first 2 years of follow-up after diagnosis and the inability to achieve remission during the same period in RA and PsA patients. Depressive or anxiety symptoms are associated with a lower likelihood of achieving remission in both RA and PsA. However, after taking into account possible overlap between depression and anxiety, only depression was associated with a lower likelihood of achieving remission. The reduced likelihood of remission is due to an increased number of tender joints, higher general health and pain scores and slightly elevated ESR and CRP levels in patients with a possible depression or anxiety disorder. Interestingly, the number of swollen joints did not differ between those with or without depression/anxiety. These results indicate that not only the presence of a possible depression at diagnosis, but also the presence of these symptoms during follow-up of the disease is important for the ability to achieve remission. Noteworthy is the fact that patients who develop a possible depression or anxiety disorder after 1 or 2 years of follow-up already score higher on the HADS at diagnosis. Moreover, patients who have a resolution of their depression/anxiety after 1 year show a larger DAS44/DAPSA improvement than patients who were persistently depressed/anxious.

We found a prevalence of depression of 20% and a prevalence of anxiety of 30% in early RA patients. In early PsA patients these prevalences were 18% and 23%, respectively. These prevalence rates are similar to previously reported prevalence rates from literature, although prevalence rates are highly dependent on the measurement instrument and cut-off value used, making proper comparison difficult [7, 34, 35].

Our results are consistent with previous literature with regard to a reduced likelihood of remission if symptoms of depression or anxiety are present in RA [12–17, 36]. In PsA there is less evidence, and in particular studies using PsA-specific composite measures to define remission in this context are lacking. Michelsen *et al.* for example, have shown that symptoms of depression or anxiety in PsA patients negatively predict DAPSA remission after 3 and 6 months, but they used a modified DAPSA with a 32 instead of a 66/68 joint count and did not differentiate between a possible depression or anxiety disorder [15]. Furthermore, Wong *et al.* have shown that depressive/anxiety symptoms reduce the probability of sustained minimal disease activity [19]. Other studies have also shown correlations between (a change in) HADS depression/anxiety scores and (a change in) DAPSA scores, but did not report on the ability to achieve DAPSA remission [37, 38]. Thus, our research adds to the current knowledge on the association between depression/anxiety and the ability to achieve remission over time in both RA and PsA.

In our study, depression was associated with an elevated ESR in RA, and anxiety was associated with an elevated CRP in PsA, although differences were small. The aforementioned is consistent with some studies showing that inflammation may play a role in the pathophysiology of depression and possibly also anxiety. Pro-inflammatory cytokines that are involved in the pathogenesis of RA and PsA, including TNF-α, IL-1 and IL-6, are also elevated in patients with a depression or anxiety disorder [39–42]. In addition, higher CRP levels have been found in the peripheral blood and cerebrospinal fluid of patients with a depression compared with healthy individuals [40]. Previous research has also shown that some anti-inflammatory therapies, including TNF inhibitors, reduce symptoms of depression in patients with an inflammatory disease [40]. However, studies on the shared pathophysiology of inflammatory arthritis and depression/anxiety have shown inconsistent results and modest effect sizes, indicating the need for more research in this area [43].

We also found that depression/anxiety were associated with more tender joints, worse general health and more pain. Symptoms of depression and anxiety may negatively impact patients’ perception of their overall health and may lower pain thresholds [41]. Previous research has shown that there is a bidirectional relationship between depression and pain [44]. Psychological symptoms can also negatively affect medication adherence and health behaviour, including physical activity and smoking [18]. Importantly, a higher tender joint count and poorer general health may affect the management of patients with RA or PsA, as it leads to a higher disease activity score. The inability to achieve remission could lead to (unnecessary) treatment intensifications, including multiple medication switches, thereby generating increased healthcare and societal costs [17, 45]. Although there are no data in RA and PsA, treatment of depression has already proven to be cost-effective in patients with diabetes mellitus and a concurrent depression [46]. In addition, cognitive behavioural therapy has shown to be effective in reducing levels of depression and anxiety in RA patients [47]. The aforementioned data underscores the importance of screening for psychological symptoms in patients with RA or PsA.

Limitations include, firstly, the inability to demonstrate causality, despite the fact that several of the Bradford Hill criteria for causation are met, and the difficulty in unravelling the direction of the relationship between a possible depression or anxiety disorder and remission [48]. Further research with crossed-lagged models could help elucidate the direction of the association found. Secondly, confounders may have influenced our results. Although adjustments were made in our analyses for possible confounders such as sex, symptom duration, smoking, baseline disease activity and enthesitis, we cannot rule out bias due to other factors that affect the ability to achieve remission. For example, deformed joints and comorbidities, including fibromyalgia, may lead to a higher DAS/DAPSA score [49]. Furthermore, depression was no longer significantly associated with remission in the adjusted sensitivity analyses with the DAS44 3-item. This shows that there is some circularity in our data and that poorer general health scores in patients with depression are an important driving factor for not achieving remission. Finally, patients who dropped out might be different from those who remained in the study. Therefore, we compared the baseline characteristics of patients who dropped out with those with a complete follow-up and found only small differences. Thus, we expect that this did not strongly influence our results, especially because we used mixed models to analyse the data.

A strength of our study is the use of the HADS, a validated instrument with subscales to assess a possible depression and anxiety disorder separately. In addition, we adjusted the analyses of depression for anxiety and vice versa, because a lot of patients scored high on both the HADS-D and HADS-A. Although the HADS cannot serve as a true diagnostic tool for a depression or anxiety disorder, it is a suitable and valid screening tool for symptoms of depression and anxiety [50]. Moreover, the HADS was administered at multiple time points during follow-up, allowing us to assess the association of depression and anxiety and remission over time. We included a relatively large sample of RA and PsA patients, making it possible to provide an overview of a possible depression or anxiety disorder in both types of inflammatory arthritis. We also used disease-specific measures to assess disease activity in both RA and PsA, making our results more easily applicable in daily practice. Finally, several sensitivity analyses were performed, including one using the HADS score on a continuous scale, which showed that even a 1-point increase on the HADS significantly reduces the likelihood of achieving remission.

In conclusion, the presence of depressive symptoms, but not anxiety, at any time during the first 2 years after diagnosis was associated with a lower likelihood of achieving remission in both RA and PsA. Having depressive symptoms is associated with more tender joints, poorer general health and more pain, as well as higher inflammation markers. Therefore, proper recognition of a depression may improve disease management of patients with RA or PsA. This could potentially result in fewer (unnecessary) treatment alterations and reduced healthcare and societal costs.

## Supplementary material

Supplementary material is available at *Rheumatology* online.

## Supplementary Material

keae621_Supplementary_Data

## Data Availability

Data are available from the corresponding author upon reasonable request.
