# Supplementary material for: Association between depression and anxiety and inability to achieve remission in rheumatoid arthritis and psoriatic arthritis
Source: Rheumatology (Oxford). 2024 Nov 6;64(5):2411–21. doi: 10.1093/rheumatology/keae621 (PMC12048054; doi:10.1093/rheumatology/keae621)
Supplement: keae621_Supplementary_Data [file keae621_supplementary_data.docx]

**Supplementary materials**

**Supplementary table S1. Completeness rate of the HADS at each timepoint**

|  | Baseline | 6 months | 12 months | 18 months | 24 months |
| --- | --- | --- | --- | --- | --- |
| RA | 94% | 82% | 78% | 48% | 60% |
| PsA | 83% | - | 76% | - | 67% |

Percentage of patients who completely filled in the HADS. The proportion is calculated based on the total number of included patients (n=425 in RA and n=442 in PsA).

*Abbreviations: HADS, Hospital Anxiety and Depression Scale; PsA, psoriatic arthritis; RA, rheumatoid arthritis.*

**Supplementary table S2. DAS44/ DAPSA change from baseline to 1 year in RA and PsA patients who are persistently (not) depressed/anxious, newly depressed/anxious or no longer depressed/anxious**

| Rheumatoid arthritis |  |  |
| --- | --- | --- |
| Baseline | **1 year** | **DAS44 change**  **baseline - 1 year (mean (sd))** |
| Depressed | Persistently depressed | 1.5 (1.3) |
| Depressed | No longer depressed | 2.1 (1.3) |
| Not depressed | Newly depressed | 1.9 (1.0) |
| Not depressed | Persistently not depressed | 1.7 (1.2) |
| Anxious | Persistently anxious | 1.8 (1.3) |
| Anxious | No longer anxious | 1.9 (1.3) |
| Not anxious | Newly anxious | 1.9 (1.4) |
| Not anxious | Persistently not anxious | 1.7 (1.1) |
|  |  |  |
| Psoriatic arthritis |  |  |
| Baseline | **1 year** | **DAPSA change**  **baseline - 1 year (mean (sd))** |
| Depressed | Persistently depressed | 5.9 (12.5) |
| Depressed | No longer depressed | 10.8 (10.6) |
| Not depressed | Newly depressed | 3.7 (10.1) |
| Not depressed | Persistently not depressed | 8.0 (11.9) |
| Anxious | Persistently anxious | 5.3 (12.6) |
| Anxious | No longer anxious | 16.1 (13.6) |
| Not anxious | Newly anxious | 8.0 (8.9) |
| Not anxious | Persistently not anxious | 7.0 (11.4) |

*Abbreviations: DAPSA, Disease Activity index for Psoriatic Arthritis; DAS44, 44-joint Disease Activity Score; HADS-A, Hospital Anxiety and Depression Scale – Anxiety subscale; HADS-D, Hospital Anxiety and Depression Scale – Depression subscale; PsA, psoriatic arthritis; RA, rheumatoid arthritis; sd, standard deviation.*

**Supplementary figure S1.** **Mean (with 95% CI) DAS44 and DAPSA score during 2 years of follow-up in RA and PsA patients with both a possible depression and anxiety disorder versus patients with only a possible depression or anxiety disorder, at any timepoint during 2 years of follow-up.**

Figure 1A shows RA patients with both a possible depression and anxiety disorder at any timepoint during 2 years of follow-up (HADS-D >7 and HADS-A >7) compared to RA patients with only a possible depression (HADS-D >7) or anxiety disorder (HADS-A >7) and RA patients without a possible depression and anxiety disorder (HADS-D ≤7 and HADS-A ≤7). Figure 1B shows PsA patients with both a possible depression and anxiety disorder at any timepoint during 2 years of follow-up (HADS-D >7 and HADS-A >7) compared to PsA patients with only a possible depression (HADS-D >7) or anxiety disorder (HADS-A >7) and PsA patients without a possible depression and anxiety disorder (HADS-D ≤7 and HADS-A ≤7).

*Abbreviations: CI, confidence interval; DAPSA, Disease Activity index for Psoriatic Arthritis; DAS44, 44-joint Disease Activity Score; HADS-A, Hospital Anxiety and Depression Scale – Anxiety subscale; HADS-D, Hospital Anxiety and Depression Scale – Depression subscale; PsA, psoriatic arthritis; RA, rheumatoid arthritis.*

**Supplementary table S3. Baseline characteristics of RA and PsA patients who dropped out and those who did not drop out.**

|  | Rheumatoid arthritis | | | |
| --- | --- | --- | --- | --- |
|  | Without data at 2 years | | With data at 2 years | |
|  | n=105 | | n=295 | |
| Demographic characteristics |  | | | |
| Age (years), mean (SD) | 54.9 | (15.1) | 53.8 | (13.8) |
| Sex (female), n (%) | 74 | (70) | 192 | (65) |
| Symptom duration (months), median (IQR) | 4 | (3-7) | 5 | (3-7) |
| Currently smoking, n (%) | 39 | (38) | 81 | (27) |
| Body mass index (kg/m^2^) , mean (SD) | 25.9 | (4.5) | 26.4 | (4.8) |
| Disease activity |  | | | |
| DAS44, mean (SD) | 3.3 | (0.9) | 3.3 | (1.0) |
| Swollen joint count (44), median (IQR) | 7 | (4-12) | 8 | (4-12) |
| Tender joint count (53), median (IQR) | 10 | (5-16) | 9 | (5-14) |
| ESR (mm/hr), median (IQR) | 22 | (12-40) | 20 | (12-36) |
| General health (VAS), median (IQR) | 53 | (32-69) | 52 | (29-67) |
|  |  |  |  |  |
|  | **Psoriatic arthritis** | | | |
|  | Without data at 2 years | | With data at 2 years | |
|  | n=75 | | n=292 | |
| Demographic characteristics |  |  |  |  |
| Age (years), mean (SD) | 48.0 | (14.6) | 51.6 | (13.2) |
| Sex (female), n (%) | 39 | (52) | 142 | (49) |
| Symptom duration (months), median (IQR) | 9 | (4-35) | 11 | (4-32) |
| Currently smoking, n (%) | 18 | (24) | 66 | (23) |
| Body mass index (kg/m^2^), mean (SD) | 28.2 | (5.5) | 28.1 | (4.9) |
| Disease activity |  |  |  |  |
| DAPSA, median (IQR) | 17 | (12-25) | 15 | (10-22) |
| Swollen joint count (66), median (IQR) | 2 | (0-3) | 2 | (1-4) |
| Tender joint count (68), median (IQR) | 3 | (1-8) | 3 | (1-7) |
| CRP (mg/L), median (IQR) | 3 | (0-8) | 4 | (1-11) |
| General health (VAS), median (IQR) | 51 | (29-71) | 44 | (23-63) |
| Pain (VAS), median (IQR) | 59 | (31-73) | 45 | (24-65) |
| Psoriasis, n (%) | 65 | (87) | 254 | (87) |
| BSA (%) in case of psoriasis, median (IQR) | 3 | (1-4) | 3 | (1-5) |
| Enthesitis, n (%) | 33 | (44) | 111 | (38) |
| LEI in case of enthesitis, median (IQR) | 2 | (1-2) | 2 | (1-3) |
| Dactylitis, n (%) | 14 | (19) | 47 | (16) |

*Abbreviations: BSA, body surface area; CRP, C-reactive protein; DAPSA, Disease Activity index for Psoriatic Arthritis; DAS44, 44-joint Disease Activity Score; ESR, erythrocyte sedimentation rate; IQR, interquartile range; LEI, Leeds Enthesitis Index; PsA, psoriatic arthritis; RA, rheumatoid arthritis; SD, standard deviation; VAS, Visual Analogue Scale.*

**Supplementary figure S2. Odds of achieving remission over 2 years of follow-up in RA and PsA patients for every point increase in HADS score at any timepoint.**

Graph 1A and B show the unadjusted and adjusted ORs with 95% CI for the likelihood of achieving remission, defined as DAS44 <1.6, during 2 years of follow-up for RA patients per point increase in the HADS score at any timepoint. Graph 1C and D show the unadjusted and adjusted ORs with 95% CI for the likelihood of achieving remission, defined as DAPSA ≤4, during 2 years of follow-up for PsA patients per point increase in the HADS score at any timepoint.

In both RA and PsA the HADS score adjusted OR was adjusted for HADS-A score (graphs 1A, 1C) or HADS-D score (graphs 1B, 1D). The fully adjusted OR was additionally corrected for sex, symptom duration and smoking. In RA we also corrected for baseline DAS and randomization strata and in PsA for the baseline DAPSA and the presence of enthesitis.

*Abbreviations: CI, confidence interval; DAPSA, Disease Activity index for Psoriatic Arthritis; DAS44, 44-joint Disease Activity Score; HADS, Hospital Anxiety and Depression Scale; HADS-A, Hospital Anxiety and Depression Scale – Anxiety subscale; HADS-D, Hospital Anxiety and Depression Scale – Depression subscale; OR, odds ratio; PsA, psoriatic arthritis; RA, rheumatoid arthritis.*

**Supplementary figure S3.** **Odds of achieving remission, measured with the DAS44 3-item, over 2 years of follow-up in RA patients with and without a possible depression or anxiety disorder at any timepoint.**

Graph S2A and B show the unadjusted and adjusted ORs with 95% CI for the likelihood of achieving remission, defined as DAS44 3-item <1.6, during 2 years of follow-up for RA patients with a possible depression or anxiety disorder, defined as a HADS >7 at any timepoint during the follow-up.

The depression/anxiety adjusted OR was adjusted for anxiety (HADS-A >7, graph 2A) or depression (HADS-D >7, graph 2B). The fully adjusted OR was additionally adjusted for sex, symptom duration, smoking, baseline DAS44-3 item and randomization strata.

*Abbreviations: CI, confidence interval; DAS44 3-item, 44-joint Disease Activity Score with 3 items (swollen joint count-44, tender joint count-53, erythrocyte sedimentation rate);* *HADS, Hospital Anxiety and Depression Scale; HADS-A, Hospital Anxiety and Depression Scale – Anxiety subscale; HADS-D, Hospital Anxiety and Depression Scale – Depression subscale; OR, odds ratio; RA, rheumatoid arthritis.*

**Supplementary figure S4**. **Odds of achieving remission over 2 years of follow-up in PsA patients with an oligo-arthritis or poly-arthritis.**

Graph S3A and B show the unadjusted and adjusted ORs with 95% CI for the likelihood of achieving remission, defined as DAPSA ≤4, during 2 years of follow-up for oligo-arthritis or poly-arthritis PsA patients with a possible depression or anxiety disorder, defined as a HADS >7 at any timepoint during the follow-up.

The depression/anxiety adjusted OR was adjusted for anxiety (HADS-A >7, graph 3A) or depression (HADS-D >7, graph 3B). The fully adjusted OR was additionally adjusted for sex, symptom duration, smoking, baseline DAPSA and the presence of enthesitis.

*Abbreviations: CI, confidence interval; DAPSA, Disease Activity index for Psoriatic Arthritis; HADS, Hospital Anxiety and Depression Scale; OR, odds ratio; PsA, psoriatic arthritis.*
